# Genomic and Pathologic Findings for *Prototheca cutis* Infection in Cat 

**DOI:** 10.3201/eid2703.202941

**Published:** 2021-03

**Authors:** Grazieli Maboni, Jessica A. Elbert, Justin M. Stilwell, Susan Sanchez

**Affiliations:** University of Guelph, Guelph, Ontario, Canada (G. Maboni);; Athens Veterinary Diagnostic Laboratory, Athens, Georgia, USA (G. Maboni, S. Sanchez);; University of Georgia, Athens (J.A. Elbert, J.M. Stilwell)

**Keywords:** *Prototheca cutis*, zoonoses, cat, whole-genome sequencing, pathology, antimicrobial susceptibility, algae, antimicrobial resistance

## Abstract

Severe nasal *Prototheca cutis* infection was diagnosed postmortem for an immunocompetent cat with respiratory signs. Pathologic examination and whole-genome sequencing identified this species of algae, and susceptibility testing determined antimicrobial resistance patterns. *P. cutis* infection should be a differential diagnosis for soft tissue infections of mammals.

*Prototheca* spp. (phylum Chlorophyta, order Chlorellales, family *Chlorellaceae*) are ubiquitous algal organisms that represent emerging infectious agents of humans and animals (*1*). Protothecosis has been increasingly reported for immunocompromised human and animal patients (*1*,*2*). At least 14 species of *Prototheca* have been recognized; 1 case of *P. cutis*–associated dermatitis in an immunocompromised man has been reported (*3*,*4*). We describe a case of *P. cutis* in a domestic cat in Georgia, USA.

In January 2020, an 11-year-old, 5.8-kg, neutered male, domestic cat was examined for sneezing, wheezing, congestion, and rhinitis. This indoor/outdoor cat was negative for feline leukemia and feline immunodeficiency viruses. The cat showed no response to treatment with steroids and cefovecin sodium (Convenia; Zoetis, https://www.zoetis.com). From June 2019 through January 2020, the nasal planum became rounded and disfigured. A biopsy sample submitted to a private diagnostic laboratory indicated a fungal infection containing organisms suggestive of *Cryptococcus* spp. Because of concerns over the zoonotic potential of *Cryptococcus* spp., the cat was euthanized and submitted for postmortem examination.

Gross reflection of the skin revealed that a locally extensive area of connective tissue and musculature overlying ≈70% of the nasal bridge and dorsal nasal planum was diffusely soft, variably tan to light orange, and mildly gelatinous (Appendix Figure 1). Microscopic evaluation revealed a severe granulomatous nodular dermatitis, panniculitis, cellulitis, pyogranulomatous osteomyelitis, and rhinitis (Figure). Disseminated throughout the nasal turbinates were numerous free and intrahistiocytic sporangia with and without endospores (Figure). Although these findings suggest previously resolved infection in the skin, subcutis, and muscle and active infection in the nasal turbinates, the initial site of infection (cutaneous vs. nasal turbinates) and disease pathogenesis could not be definitively determined (Appendix).

**Figure:**
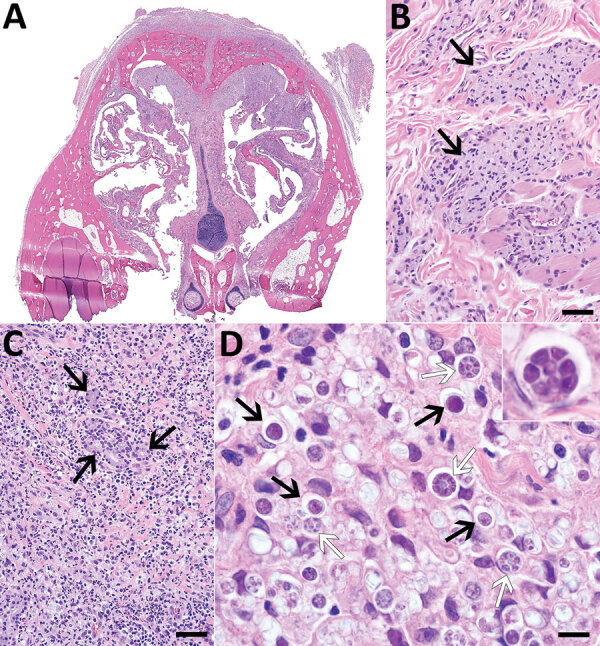
Histologic lesions associated with protothecosis caused by *Prototheca cutis* in a cat. A) Subgross cross-section of the nasal turbinates showing marked expansion of the nasal epithelium and overlying subcutaneous tissue. B) Epithelioid macrophages (arrows) with abundant, intracytoplasmic, gray material multifocally dissecting through subdermal collagen and musculature. Hematoxylin and eosin (H&E) stain; scale bar indicates 50 μm. C) Submucosal glands (arrows), markedly displaced by myriad macrophages, neutrophils, lymphocytes, plasma cells, and algal sporangia. H&E stain; scale bar indicates 50 μm. D) Algal sporangia (black arrows), which sometimes endosporulate (white arrows), producing up to 8 endospores (inset). H&E stain; scale bar indicates 10 μm and does not apply to inset.

Fungal culture yielded white colonies growing in the presence and absence of light at 30°C (Appendix Figure 2). Cytologic examination revealed colonies of round cells with internal septations and thick walls resembling sporangia and endospores, which were identified with lactophenol cotton blue stain (Appendix Figure 2). Sporangia were gram positive, although they appeared to be unevenly stained (Appendix Figure 2). Genus and species were not identified by matrix-assisted laser desorption ionization/time-of-flight mass spectrometry and GEN III Microbial identification (Biolog, https://www.biolog.com). Partial sequencing of the internal transcribed spacer region and the D1/D2 region of the 28S rRNA gene yielded sequences 96% and 99% homologous to those from *P. cutis* available in BLAST (https://blast.ncbi.nlm.nih.gov) and CBS-KNAW (https://www.knaw.nl, currently Westerdijk Fungal Biodiversity Institute) databases.

Because the mitochondrial *cytb* gene potentially represents a new standard method for identifying *Prototheca* species (*5*), we performed whole-genome sequencing to investigate *cytb* as well as other genes by using Illumina MiSeq (https://www.illumina.com) (Appendix). The nuclear genome was 19,237,076-bp long, and the plastid genome was 51,673-bp long, which corresponds to genome sizes obtained from sequencing of *P. cutis* JCM15793 strain ATCC PRA-138 (https://www.atcc.org) (*6*). We submitted the genome assembly to GenBank (accession no. JABBYS000000000). The in silico–targeted gene alignment revealed genes homologous with available *P. cutis* sequences including *cytB* (99.8%, accession no. MT363977), chloroplast genome DNA (99.54%), 18S rRNA gene (100%, accession no. MT360051), ITS (99.12%, accession no. MT359908), and 28S rRNA (D1/D2 domain) (100%, accession no. MT360265), which were deposited in GenBank.

We performed susceptibility testing for antifungal and antimicrobial drugs because both have been used against *Prototheca* spp. infections in animals (*7*,*8*). The isolate showed high MICs for fluconazole and itraconazole and a low MIC for amphotericin B (Table). Resistance to fluconazole and susceptibility to amphotericin B are well recognized for other *Prototheca* species (*9*); however, resistance to itraconazole could be unique to *P. cutis*. MICs were high for most antimicrobial drugs, including cefovecin, which had been unsuccessful in treating the cat (Table). Oxacillin, pradofloxacin, and trimethoprim/sulfamethoxazole inhibited growth at the lowest concentrations, indicating in vitro sensitivity to these drugs. We further investigated whether resistance genes from the whole-genome sequence corresponded to phenotypic resistance. We used the Comprehensive Antibiotic Resistance Database (https://card.mcmaster.ca) to identify genes conferring resistance to β-lactams, tetracyclines, aminoglycosides, chloramphenicol, and vancomycin. Consistent with MICs, no resistance genes corresponded to pradofloxacin and trimethoprim/sulfamethoxazole (Table). The MIC data provided here may be helpful for establishing future clinical breakpoints for *Prototheca* spp.

**Table:** Antimicrobial and antifungal MICs and resistance genes identified by whole-genome sequencing of *Prototheca cutis* isolated from a nasal lesion in a cat*

Class	MIC, μg/mL	AMR genes
Antimicrobial testing†		
β-lactams		*Oxa-168, NmcA, OXA-198, IMP-8*
Amoxicillin/clavulanate	8	
Ampicillin	>8	
Oxacillin	<0.25	
Penicillin G	8	
Cefazolin	>4	
Cefovecin	>8	
Cefpodoxime	>8	
Cephalothin	>4	
Imipenem	4	
Tetracyclines		*tet(31)*
Doxycycline	>0.5	
Minocycline	1	
Tetracycline	>1	
Quinolones		None found
Enrofloxacin	>4	
Marbofloxacin	2	
Pradofloxacin	<0.25	
Aminoglycosides		*AAC(6')-Ij, AAC(* *3* *)-Xa, AAC(* *3* *)-VIIIa*
Amikacin	>32	
Gentamicin	8	
Lincosamides: clindamycin	4	None found
Macrolides: erythromycin	>4	None found
Rifamycins: rifampin	>2	None found
Nitrofurans: nitrofurantoin	>64	None found
Phenicols: chloramphenicol	>32	*catB10*
Sulfonamides: trimethoprim/sulfamethosoxazole	<2	None found
Vancomycin	>16	*vanA, vanRO*
Antifungal testing‡		Not investigated
Azoles		
Fluconazol	>256	
Itraconazol	>32	
Polyenes: amphotericin B	0.19	

*AMR genes were identified by using the Comprehensive Antibiotic Resistance Database (https://card.mcmaster.ca). Interpretation of MICs was not included in this table because clinical breakpoints for *Prototheca* spp. are not available. AMR, antimicrobial resistance. †Antimicrobial susceptibility testing was performed by using a Trek Sensititre Gram Positive panel (TREK Diagnostic Systems, http://www.trekds.com). ‡Antifungal susceptibility testing was performed by using MIC test strips (Liofilchem, https://www.liofilchem.com).

Our primary concern with regard to this case was determining the zoonotic potential of the agent, which was initially misdiagnosed as *Cryptococcus* spp. Although *Prototheca* spp. are widely considered to be zoonotic agents, reports of definitive cases of zoonotic transmission are lacking in the literature. Zoonotic transmission from bovids is thought to occur via consumption of contaminated milk (*10*). The zoonotic potential of *P. cutis* is unclear; infectivity is probably similar to that of other *Prototheca* spp.

Our report of *P. cutis* isolation from a cat reinforces protothecosis as an emerging infectious disease of humans and animals. We emphasize the potential of *P. cutis* to infect presumably immunocompetent hosts. The veterinary and human medical communities should be aware of the unusual clinical, pathologic, and microbiological manifestations of protothecosis.

Appendix Detailed gross and histopathologic methods and findings associated with diagnosis of *Prototheca cutis* infection in a cat.
