# Supplementary material for: Genomic and Pathologic Findings for Prototheca cutis Infection in Cat
Source: Emerg Infect Dis. 2021 Mar;27(3):979–82. doi: 10.3201/eid2703.202941 (PMC7920651; doi:10.3201/eid2703.202941)
Supplement: Appendix — Detailed gross and histopathologic methods and findings associated with diagnosis of Prototheca cutis infection in a cat. [file 20-2941-Techapp-s1.pdf]

Article DOI: https://doi.org/10.3201/eid2703.202941

Genomic and Pathologic Findings for *Prototheca cutis* Infection in Cat

Appendix

Gross and histopathologic descriptions, fungal culture, phenotypic identification, antimicrobial and antifungal susceptibility testing, Sanger sequencing, and whole genome sequencing.

1. Gross and histopathologic descriptions

Grossly, the dorsal nasal planum and nasal bridge was irregularly rounded, enlarged, and bulging with simple interrupted sutures closing the previous biopsy site. A locally extensive area of connective tissue and musculature overlying ≈70% of the nasal bridge and dorsal nasal planum was diffusely soft, variably tan to light orange, and mildly gelatinous. The subjacent nasal turbinates were mildly swollen, red, and soft. Histologic evaluation of the dermis and subcutis overlying the nasal bridge revealed a multifocal to coalescing, nodular, densely cellular population composed predominantly of epithelioid macrophages filled with intracytoplasmic amorphous, gray material. The inflammatory population extended through the underlying bone and disrupted and effaced the submucosa and mucosa of ≈70% of the dorsal nasal turbinates, resulting in multiple, locally extensive areas of ulcerated mucosa, displacement of submucosal glands, and turbinate bone loss. The macrophages in the nasal turbinates were admixed with few degenerate and non-degenerate neutrophils, lymphocytes, plasma cells, multinucleated giant cells, and rare eosinophils and Mott cells. Scattered throughout the inflammation were numerous, extracellular and intrahistiocytic, round to oval, 8–20 μm diameter algal sporangia that had a clear, 2–4 μm thick wall and contained either a central basophilic nucleus or multiple (2–8) wedge-shaped, radially arranged endospores. Algal cell walls were strongly positive in Periodic acid–Schiff and Grocott-Gomori's methenamine silver stains. Luminal spaces within the nasal cavity contained variable aggregates of the previously described inflammatory population and free algal sporangia admixed with scant hemorrhage and mucin. Variable amounts of necrosis, fibrin, and scant hemorrhage were scattered throughout all affected regions. Other histologic

findings unrelated to algal infection included segmental, lymphoplasmacytic and eosinophilic enteritis, renal cortical cysts with lymphocytic interstitial nephritis, nodular adrenal cortical hyperplasia, and presumptive Sarcocystosis within the skeletal and cardiac muscle.

The definitive pathogenesis of protothecosis is unknown. One possible route of infection is traumatic cutaneous inoculation from a penetrating injury, which is consistent with previous reports of Protothecosis in cats (*1*). Macrophages within the dermis, subcutis, and bone contained intracytoplasmic gray material presumptively representing phagocytosed sporangial walls post endosporulation, which was seen actively occurring within in the nasal turbinates. Given the length of time between initial infection and presentation to necropsy, the findings suggest the inoculating wound and initial site of infection could have been removed via biopsy or resolved with subsequent dissemination of the pathogen deeper into the subcutis and nasal turbinates. Another possibility reflects infection via the nasal cavity and turbinates with extension of inflammation dorsally through the overlying bone into the subcutis and dermis. This latter possibility is considered less likely given the monomorphic population of debris-laden macrophages and the lack of sporangia or endospores within the dermis, subcutis and nasal bone that suggest previous resolution of infection at these sites rather than active spread. Further, chronic tissue remodeling would be expected within the nasal turbinates given the protracted disease progression of this case if the turbinates were the initial site of infection.

2. Fungal culture and phenotypic identification

Nasal turbinate tissue was cultured on Sabouraud dextrose agar (BD BBL, Franklin Lakes, NJ, USA), and on 5% sheep blood agar (Remel, San Diego, CA, USA) for 3 days of incubation at 25°C and 30°C. Lactophenol cotton blue and Gram stains were performed on isolated colonies. Matrix-assisted laser desorption ionization time of flight (MALDI-TOF) mass spectrometry analysis (VITEK MS – bioMérieux, Marcy-l’Étoile, France), and GEN III Microbial ID (Biolog, Hayward, CA, USA) analysis were performed as an attempt of genus/species identification. No ID was obtained, but the isolate assimilated: D-glucose, D-trehalose, acetic acid, weakly D-galactose, L-arabinose, glycerol, fructose and mannose.

3. Antimicrobials and antifungal susceptibility testing

Antimicrobial susceptibility testing was performed to determine the MIC (MIC) of 24 antimicrobials using Trek Sensititer COMPGP1 Gram positive panel (Trek Diagnostic Systems,

Cleveland, OH), according to manufacturer`s instructions. A suspension at 0.5 McFarland (10 ml) was used to inoculate the plate. Results were read visually after 24h of incubation at 30°C. Antifungal susceptibility testing was performed to determine the MIC of amphotericin B, fluconazole and itraconazol using Liofilchem MIC test strips with increasing concentration gradients of the antifungals (Liofilchem, Waltham, MA, USA), according to Clinical Laboratory Standards Institute (CLSI) document M27Ed4. Results were read visually after 24 and 48h of incubation at 30°C. All susceptibility tests were performed in duplicate. No universally accepted MIC interpretations of antimicrobials and antifungals specific for *Prototheca* species are available.

4. Sanger sequencing

Conventional pan-fungal PCR was performed targeting the internal transcribed spacer (ITS) region as well as the D1/D2 region of the large subunit of the 28S ribosomal RNA gene. *Aspergillus niger* ATCC 16404 was used as a positive control. PCR products were purified using the QIAquick PCR purification kit (Qiagen, Hilden, Germany) and sequenced by Sanger method at the AVDL (SeqStudio Genetic Analyzer, Thermo Scientific, Waltham, USA). BLAST (http://www.ncbi.nlm.nih.gov/BLAST) and the CBS-KNAW fungal database (http://www.westerdijkinstitute.nl/collections/) were used to identify related fungal sequences. Due to the rarity of *P. cutis* reports in the literature, we repeated the DNA extraction, PCR assays and sequencing. The second analysis yielded the same results and confirmed the presence of *P. cutis*.

5. Whole genome sequencing

Whole genome sequencing was carried out using the MiSeq platform (Illumina, San Diego, California), at the Athens Veterinary Diagnostic Laboratory (University of Georgia). DNA was obtained from the isolate grown on Sabouraud dextrose agar using a commercial kit, according to manufacturer’s instructions (ZR fungal/bacterial DNA MiniPrep, Zymo Research, Irvine, CA, USA). DNA concentration (17 ng/ml) was determined using a Qubit 2.0 fluorometer with a double-stranded DNA assay kit (Thermo Scientific, Waltham, MA, USA). A paired-end library with approximate insert size of 151 bp was prepared using Nextera DNA Flex library preparation kit (Illumina), and MiSeq reagent Kit v3 (150 cycles) chemistry according to the manufacturer’s protocol. The libraries were quality checked using a capillary electrophoresis device (QIAxcel, Qiagen).

The following *in silico* analyses were performed using the GenomeTrakr Network, GalaxyTrakr 1909 (https://www.galaxytrakr.org). The paired reads were first quality checked using Trimmomatic to remove adapters (Galaxy Version 0.36.4) and FastQC to select reads with Phred scores >30 (Galaxy Version 0.72+galaxy1). Assembly was performed using SPAdes software (Galaxy Version 3.12.0+galaxy1). The quality of assemblies was assessed using QUAST (Galaxy Version 5.0.2+galaxy0). Automated nuclear genome annotation was performed using Augustus (http://bioinf.uni-greifswald.de/augustus/submission.php), using *Chlamydomonas reinhardtii* as a reference species. Illumina MiSeq generated 2,008,194 paired-end reads with a genome coverage of ≈15x. The N50 read length was 5,846 bp and GC content was 61.07%. Plastid genome sequences were identified using BLAST against the chloroplast genome sequence of *P. cutis* ATCC PRA-338 (accession number: AP018373.1). Genome assemblies were imported into Geneious Prime (version 2020.1, https://www.geneious.com/prime/) for *in-silico* targeted gene evaluation. *P. cutis* genes of interest had their DNA sequences extracted from the whole genome assemblies. For that, sequences of *P. cutis* available in GenBank were aligned with whole genome assemblies from this study using Progressive Mauve aligner. These sequences included: ATCC PRA-338 partial *cytB* (599 bp, accession number MF163453.1), partial 28S rRNA (D1/D2 domain, 582 bp, accession AB470469.1), complete 18S rRNA gene (1,816 bp, accession MF163514.1), complete ITS (785 bp, accession KP898389.1), and complete plastid DNA genome (51,673 bp, accession AP018373.1).

Antimicrobial resistance genes were identified using the Comprehensive Antibiotic Resistance Database (CARD) with criteria selected as: perfect and strict hits only, excluding nudge of loose hits to strict, and high quality/coverage sequences (>70%) (https://card.mcmaster.ca/ accession 4/04/2020). The resistance genes were compared with phenotypic resistance to antimicrobials. As an attempt to identify antifungal resistance genes, we used the whole genome assemblies as input in the Mycology Antifungal Resistance Database (MARDY) (http://mardy.dide.ic.ac.uk/ accession 4/21/2020); however, no resistance genes were identified likely because of the lack of *Prototheca* in this database.

Reference

1. Masuda M, Jagielski T, Danesi P, Falcaro C, Bertola M, Krockenberger M, et al. Protothecosis in dogs and cats—new research directions. Mycopathologia. 2020 Nov 18 [Epub ahead of print]. PubMed

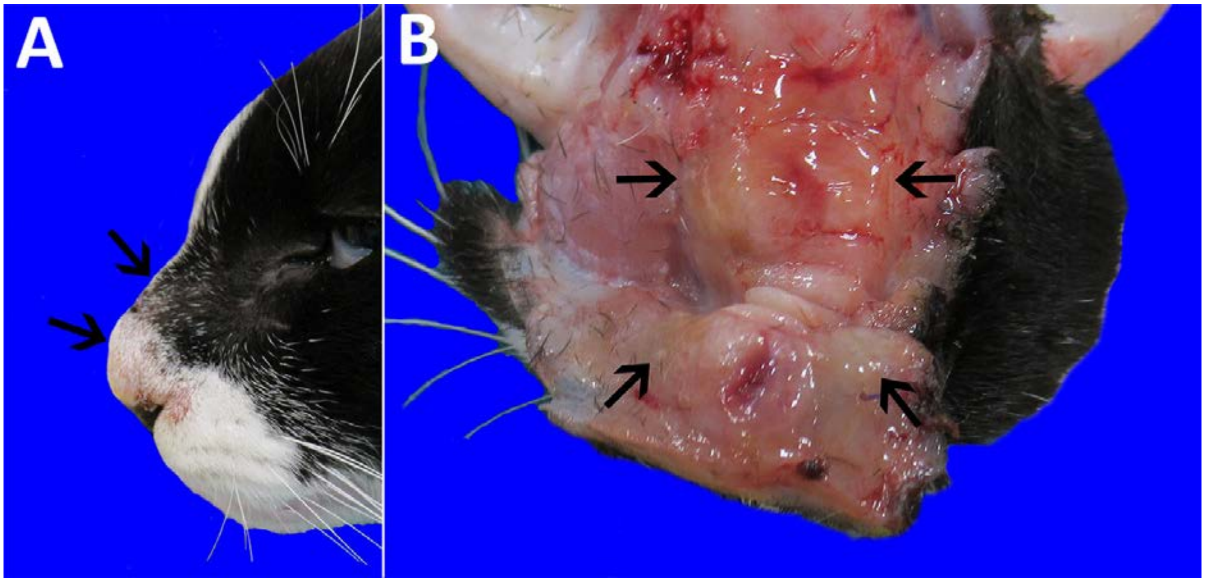

Appendix Figure 1. Gross lesions associated with protothecosis due to *Prototheca cutis* in a cat. A) Lateral view showing swelling and rounding of the nasal bridge. B) Dorsal view showing soft, tan to light orange, and mildly gelatinous connective tissue and musculature overlying the nasal bridge and dorsal nasal planum.

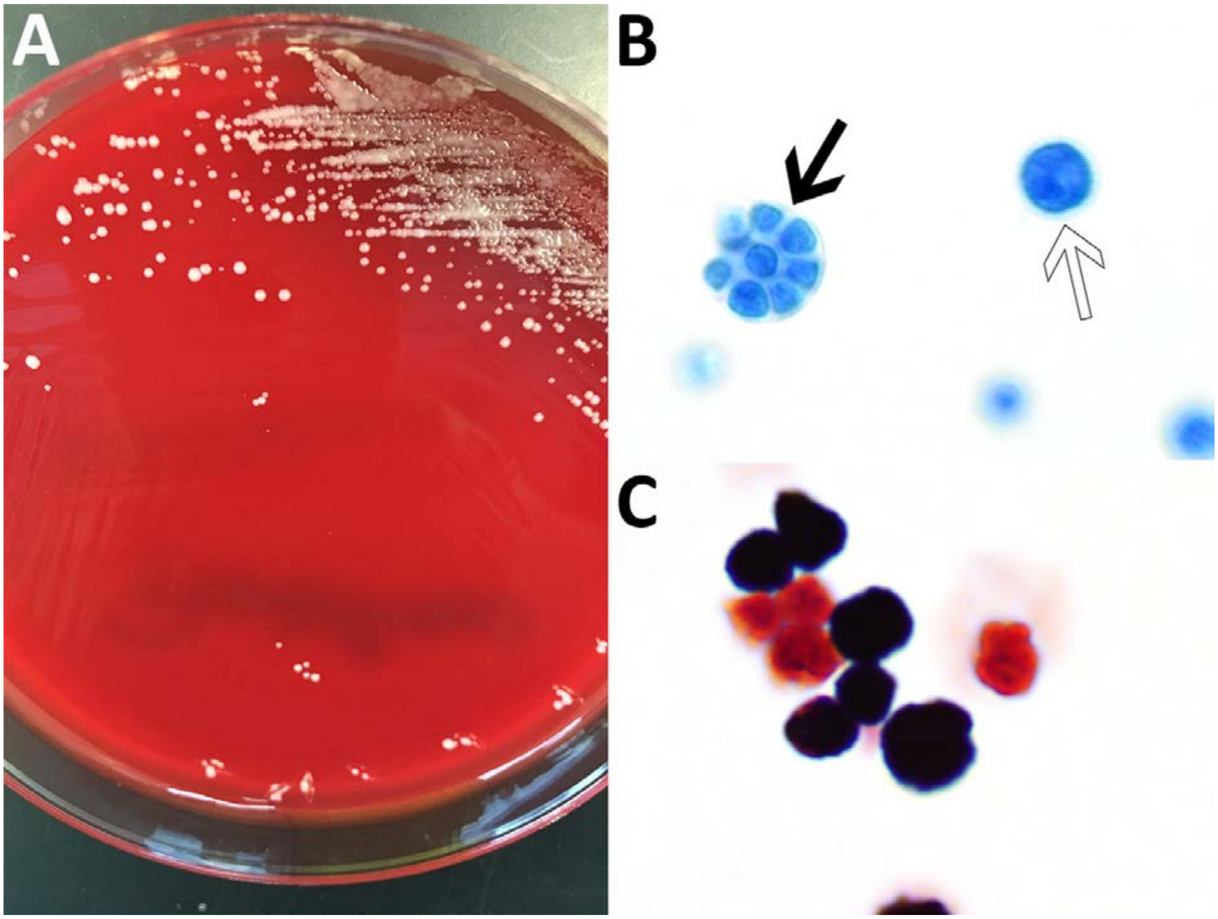

Appendix Figure 2. Culture results associated with protothecosis due to *Prototheca cutis* in a cat. A) Smooth and white colonies of *P. cutis* on sheep blood agar after incubation at 30°C for 72 h. B) Lactophenol cotton blue stain of *P. cutis* colonies showing the characteristic internal septations and thick cell walls of the sporangia, containing endospores (black arrow). During asexual reproduction, sporangiospores are released through a split which develops in the sporangial wall (arrowhead). Bar = 10 µm. C) Microscopic features of *P. cutis* revealing ovoid gram-positive cells, which are usually unevenly stained and do not show the characteristic internal septations. Bar = 10 µm.
